# Association of immune-inflammation indexes with incidence and prognosis of diabetic nephropathy: a systematic review and meta-analysis

**DOI:** 10.3389/fendo.2025.1532682

**Published:** 2025-08-18

**Authors:** Yijue Wang, Yan Liu, Wenling Gu, Boyu Cai, Min Lei, Yingyu Luo, Nannan Zhang

**Affiliations:** ^1^ National Center for Birth Defect Monitoring, Key Laboratory of Birth Defects and Related Diseases of Women and Children, Ministry of Education, West China Second University Hospital, Sichuan University, Chengdu, Sichuan, China; ^2^ West China School of Stomatology, Sichuan University, Chengdu, Sichuan, China; ^3^ Key Laboratory of Birth Defects and Related Diseases of Women and Children, Ministry of Education, Sichuan University, Chengdu, Sichuan, China; ^4^ Scaled Manufacturing Center of Biological Products, Management Office of National Facility for Translational Medicine, West China Hospital of Sichuan University, Chengdu, China; ^5^ College of Life Sciences, Sichuan University, Chengdu, Sichuan, China

**Keywords:** diabetic nephropathy, immune-inflammation index, biomarkers, diagnostic techniques, prognosis, meta-analysis, systematic review

## Abstract

**Introduction:**

The significance of immune-inflammation indexes in diabetic nephropathy (DN) was assessed in this meta-analysis to offer guidance for clinical diagnosis and treatment for DN.

**Methods:**

We performed a meta-analysis on the association between immune-inflammation indexes and the incidence and prognosis of DN, specifically focusing on the neutrophil-to-lymphocyte ratio (NLR), platelet-to-lymphocyte ratio (PLR), monocyte-to-lymphocyte ratio (MLR), systemic immune-inflammation index (SII), and systemic inflammation response index (SIRI). We thoroughly searched PubMed, Web of Science, Embase, and Cochrane from inception to September 2024. The statistical analysis was performed using R 4.2.3 software.

**Results:**

56 studies were ultimately included, comprising 50 that examined the association between DN incidence and immune-inflammation indexes and 8 that examined the association between DN prognosis and immune-inflammation indexes. The levels of NLR, MLR, PLR, and SII were significantly higher in DN patients than in non-DN ones. Besides, high NLR, MLR, SII, and SIRI were associated with elevated incidence of DN. Moreover, the high NLR group was more prone to a poor prognosis than the low NLR group (OR: 1.372, 95% CI: 1.160-1.624).

**Conclusions:**

Immune-inflammation indexes can, to a certain extent, serve as a biomarker to predict the occurrence of DN. In addition, high NLR has a potential association with the occurrence of poor prognosis in DN.

## 1 Introduction

Diabetic nephropathy (DN) is one of the most prevalent and severe chronic microvascular complications of diabetes (1), clinically characterized by progressive renal hypofunction, with or without proteinuria, which affects approximately 25%-40% of diabetes mellitus patients (2). The global incidence of DN constantly rises, and it is reported that its incidence is expected to increase by about 50% over the next two decades, resulting in approximately 783 million patients worldwide (3). DN has nowadays been the major cause of chronic kidney disease (CKD) and end-stage renal disease(ESRD) requiring dialysis or transplantation, placing a heavy burden on the economy and public health systems globally (4). However, DN has often been in an intermediate to advanced stage once persistent proteinuria develops due to insidious and progressive onset, greatly increasing the difficulty of treatment and leading to a poor prognosis (5). Moreover, a radical cure for DN remains an unfulfilled medical requirement, so early screening and detection and timely control of DN are critical to patients’ quality of life and prognosis.

Chronic inflammation, inflammation, and oxidative stress play important roles in DN progression (6, 7). As confirmed by several studies, inflammatory factors including chemokines, TNF-α, adhesion molecules, and interleukins (8, 9) are significant contributors to the development of DN (6, 10). Inflammatory factors cause inflammatory infiltration and injury in renal tissue by participating in the recruitment and infiltration of inflammatory cells, and affect the structure and function of the kidney by promoting the proliferation of renal mesangial cells and the deposition of extracellular matrix. However, these cytokines are high in cost of analysis and thus are not routinely used in clinical practice. Novel immune-inflammation indexes developed based on hemogram parameters (neutrophil/lymphocyte/platelet counts) commonly include platelet-to-lymphocyte ratio (PLR), neutrophil-to-lymphocyte ratio (NLR), monocyte-to-lymphocyte ratio (MLR), systemic immune-inflammation index (SII) [(neutrophil count × platelet count)/lymphocyte count], systemic inflammation response index (SIRI) [(neutrophil count × monocyte count)/lymphocyte count] (11). These indexes provide a more sensitive picture of the immune-inflammation balance in the body than a single blood cell count (12). Moreover, immune-inflammation indexes that are simple in calculation and easy to access have been applied as new markers for systemic inflammatory response in a variety of diseases and are also recognized as independent predictors for incidence, mortality, and long-term survival rate in many clinical settings (13–15). The association of immune-inflammation indexes with DN remained controversial in previous retrospective studies. A paired study found no correlation between NLR and DN among 1192 patients with Type 2 diabetes mellitus (T2DM) (16), whereas more studies have suggested the correlation of NLR with DN (12, 17). One of the possible reasons for this contradiction is an insufficient sample size of a single study, making the statistical validity questionable. Therefore, the association of immune-inflammation indexes with DN requires further evidence-based study.

Liu et al. described NLR’s correlation with DN in a meta-analysis (2), but they failed to convincingly clarify the relationship between NLR and DN grade due to the limited studies included (2). Therefore, this meta-analysis was conducted on all available studies on the association of immune-inflammation indexes with the incidence and prognosis of DN. This study intends to assess the value of immune-inflammation indexes for predicting DN incidence, progression, and prognosis, hoping to offer references for decision-making of clinical diagnosis and treatment of DN. Meanwhile, timely monitoring of the changes in these indexes in T2DM patients may also offer new ideas and methods for DN prevention and treatment.

## 2 Materials and methods

### 2.1 Search strategy

This meta-analysis was performed following the statement of PRISMA (Preferred Reporting Items for Systematic Reviews and Meta-Analyses). We searched PubMed, Web of Science, Embase, and Cochrane from inception to September 2024. Medical subject headings and keywords were used: Diabetic Kidney Disease, Lymphocytes, Monocytes, and Neutrophils. The search strategy and search terms are provided in **Supplementary Table S1**. This meta-analysis was registered with PROSPERO (CRD42024578732).

### 2.2 Study selection

Inclusion criteria: i) case-control studies with the expression profile of blood-derived immune-inflammation indexes (NLR, PLR, MLR, SII, and SIRI) in T2DM patients with or without DN; ii) case-control studies reporting odds ratios (ORs), as well as studies presenting sufficient data to compute ORs or reporting ORs derived from multivariable analyses; iii) cohort studies reporting the incidence rate or prognosis of T2DM patients with DN under different levels of immune-inflammation indexes over the follow-up period; iv)T2DM patients diagnosed with DN based on the criteria established by the American Diabetes Association (18). Notably, the outcome (prognosis) of DN was described as either of the following: a) all-cause mortality, b) cardiovascular mortality, c) rapid eGFR decline, or d) renal failure. Decreasing in eGFR of ≥ 25% from baseline during the follow-up was defined as an eGFR decline.

Exclusion criteria: i) duplicate publications; ii) animal or cell studies; iii) editorials, letters, meeting abstracts, and comments; iv) systematic reviews or meta-analyses.

The references of original studies were manually searched. Two researchers (WYJ and LY) were responsible independently for the study screening and selection, and the results were checked by a third researcher (ZNN).

### 2.3 Data extraction and quality evaluation

Two researchers (WYJ and LY) extracted the following data independently: i) study characteristics: author, study name and year, study period, region, and study design; ii) patient demographics: population, DN diagnostic criteria, immune-inflammation indexes, sample size, gender distribution, age, HbA1c, Albuminuria (microalbuminuria, macroalbuminuria), eGFR, and duration of disease; iii) pooled OR with 95% Cl for the association of immune-inflammation indexes with DN; iv) values of immune-inflammation indexes (mean ± standard deviation) in T2DM patients with or without DN. Albuminuria including microalbuminuria and macroalbuminuria defined as 30 mg/g ≤albumin-to-creatinine ratio (UACR)≤ 300 mg/g or UACR > 300 mg/g.

The modified Newcastle-Ottawa Scale (NOS) (19) was used for quality evaluation from selection, comparability, and exposure/outcome. Each study was rated as low (0–4), moderate (5–6), and high quality (7–9).

### 2.4 Statistical analysis

**Figure 1:**
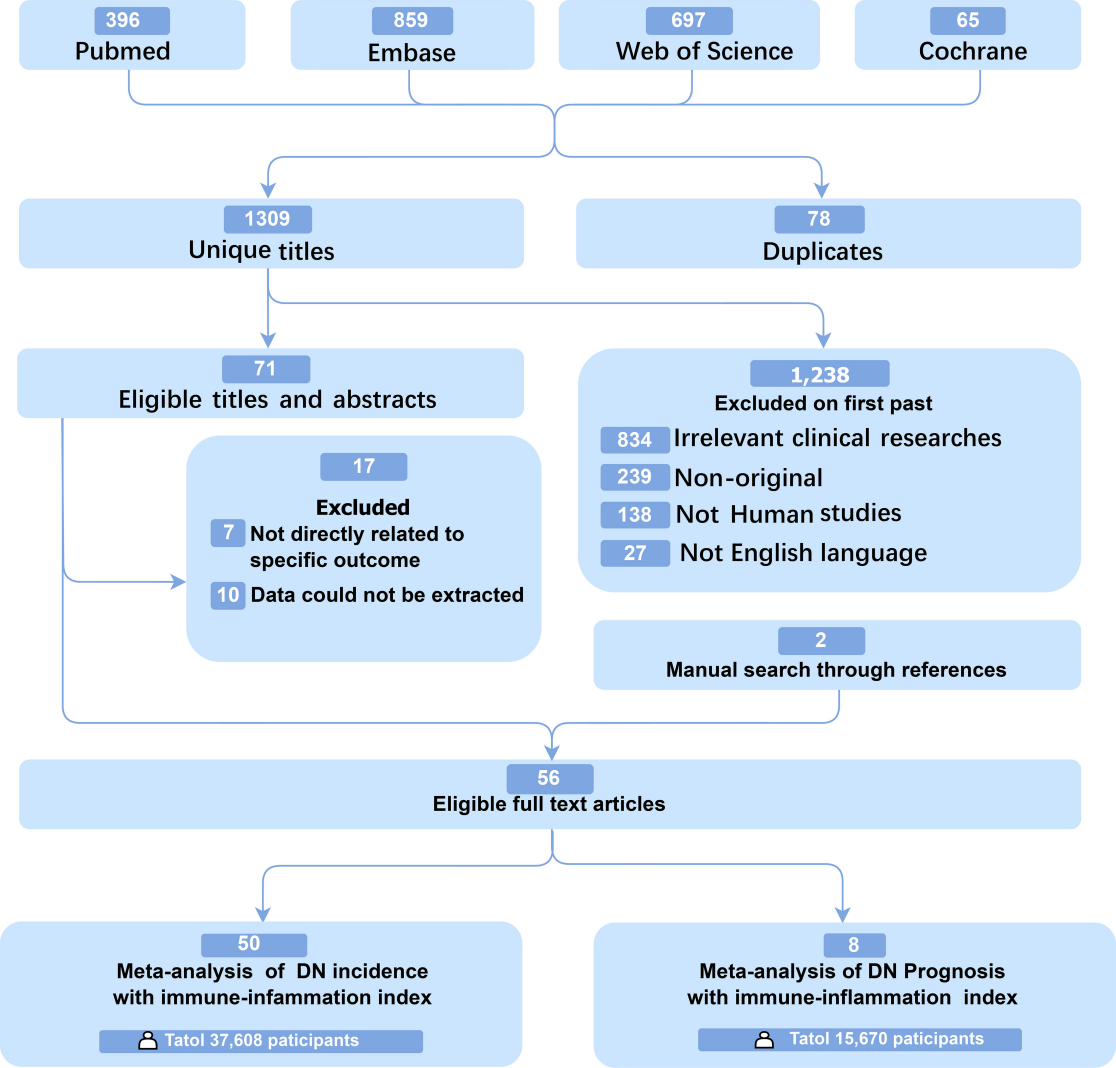
Flow diagram based on the Preferred Reporting Items for Systematic Reviews and Meta-Analysis showing the method of identifying trials and reasons for exclusion.

This study reported the incidence and prognosis of DN (**Figure 1**). Categorical and continuous variables that satisfied the inclusion criteria were documented. Outcomes were reported as the pooled OR, SMD, and 95% CI, and the interquartile range or median was transformed into mean ± SD by a standard approach (20, 21). The I² test was performed for heterogeneity, and P<0.1 and I^2^>50% were indicative of high heterogeneity, and then a random-effects model was utilized for all analyses. Subgroup analyses were conducted based on the region, age, sample size, HbA1c, albuminuria, eGFR, and duration of disease to explore the source of heterogeneity. Sensitivity analyses were performed on the overall results, which were not conducted if the number of studies was limited (less than three). Publication bias was explored by Egger’s tests and funnel plots, which were not conducted if the number of studies was limited (less than ten). R 4.2.3 was adopted for statistical analyses.

## 3 Results

### 3.1 Study characteristics

Initially, 2017 studies were retrieved from the databases, and two studies (16, 22) were obtained by manual search. The article filtering process is shown in **Figure 1**. Ultimately, this meta-analysis included 56 eligible studies (12, 16, 17, 22–74). Among them, 48 studies (12, 17, 24–27, 29, 31–58, 60–65, 67–71, 73, 74) only reported incidence-related data, six (22, 23, 30, 59, 66, 72) only presented prognosis-related data, and two (17, 28) provided both. There were 47 case-control studies, eight cohort studies, and one case-control plus cohort study. 20 studies were conducted in China (12, 16, 29, 30, 32, 35, 36, 40–42, 52–54, 56, 65, 67, 68, 72–74), 11 in Turkey (24, 33, 34, 46, 49, 51, 58, 63, 64, 70, 71), 10 in India (17, 26, 27, 38, 45, 50, 60–62, 69), four in US (22, 37, 55, 72), three in Japan (23, 48, 59), and one article from each of the other countries. NLR was investigated in 47 studies (16, 17, 22–28, 30–36, 38–42, 44–50, 52–62, 64–67, 70, 72–74), PLR in 15 studies (28, 29, 41, 43, 55, 71), MLR in six studies (24, 28, 32, 34, 44, 45, 47, 52, 54–56, 58, 62, 69, 71), SII in eight studies (12, 22, 37, 52, 55, 62, 63, 68), and SIRI in two studies (12, 55). Notably, 15 studies (12, 22, 24, 28, 32, 37, 44, 45, 47, 52, 54–56, 58, 62) reported the association of immune-inflammation indexes with DN (**Supplementary Table S2**). In addition, the NOS scores of the included studies were 6-8 (**Supplementary Tables S3**&**4**), suggesting moderate to high quality.

### 3.2 Association of immune-inflammation indexes with DN incidence: meta-analysis

#### 3.2.1 Differences in NLR levels between DN and non-DN patients

**Figure 2:**
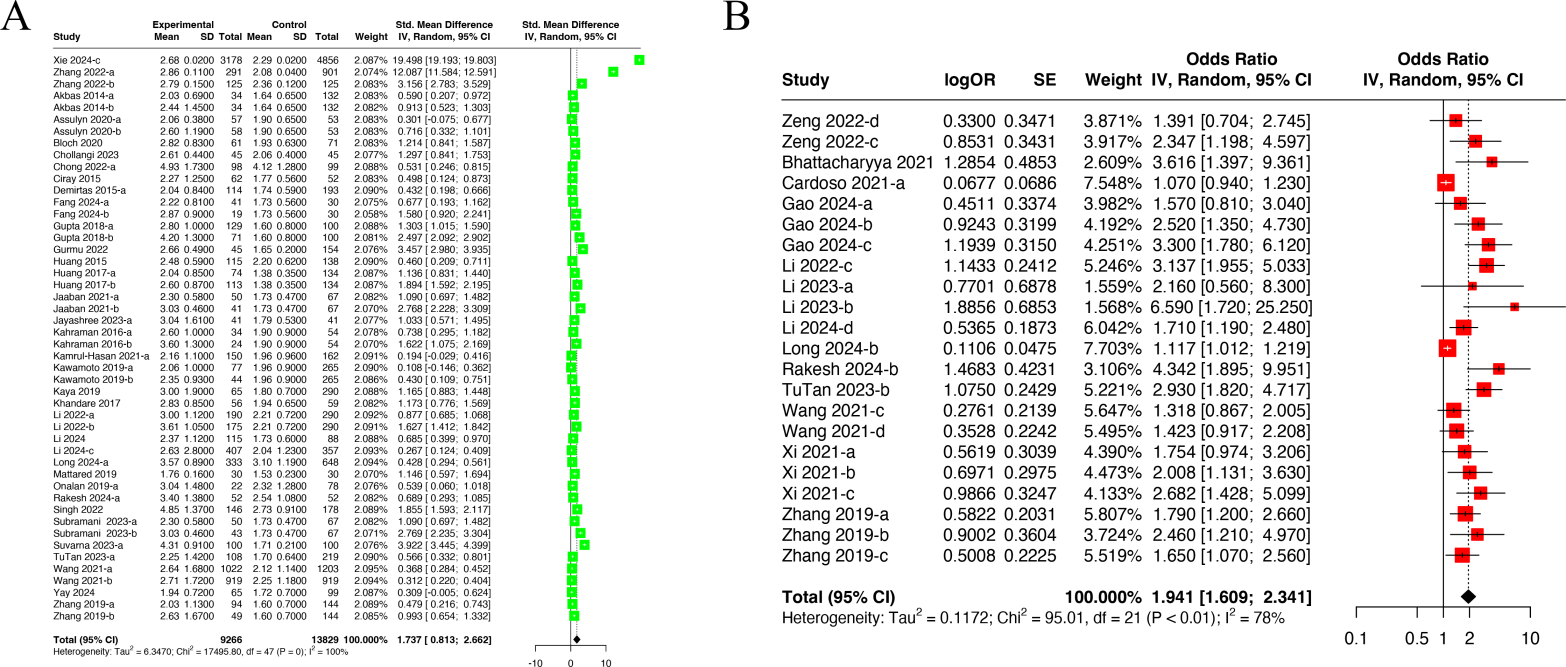
Forest plots illustrating the outcomes of the connection between NLR and DN incidence. **(A)** Forest plots for NLR levels in DN patients; **(B)** Forest plots for incidence of DN in high NLR and low NLR. NLR, Neutrophil-to-Lymphocyte Ratio.

The meta-analysis covered 48 datasets from 35 studies (16, 17, 22, 24, 25, 27, 31–35, 38–42, 44–50, 52–54, 56–58, 60–62, 64, 65, 70, 73) containing 9,266 DN patients and 13,829 non-DN patients (control group) (**Figure 2A**). A random-effects model was adopted due to significant heterogeneity among the included studies (I^2^ = 100.0%, P<0.001). The level of NLR was higher in DN patients than in non-DN patients (SMD=1.737, 95% CI: 0.813-2.662).

Subgroup analyses revealed no significant difference in heterogeneity (**Table 1**). The predictive value of NLR as a continuous variable for DN vanished in subgroups with age greater than 60 years, sample size greater than 310, and eGFR less than 90 mL/min/1.73 m^2^, however, it still had statistical significance in other subgroups.

**Table 1:** Subgroup analysis of the relationship between NLR (Continuous & Categorical), PLR (Continuous) with DN based on Region, Age, Sample size, HbA1c, albuminuria, eGFR, and Disease duration year.

Subgroup	NLR Categorical			NLR continuous			PLR continuous		
	Study	OR [95%CI]	I^2^	Study	SMD [95%CI]	I^2^	Study	SMD [95%CI]	I^2^
Total	22	1.941[1.609; 2.341]	78.0%	48	1.737[0.813; 2.662]	100.0%	17	0.637[0.307; 0.967]	93.0%
Region									
Asia	20	1.895[1.572; 2.284]	79.0%	47	1.347[0.813; 2.662]	95.2%	15	0.589[0.231;0.948]	92.8%
America	2	3.779[0.003; 4518.092]	24.0%	1	19.498[19.193;19.803]	–	2	1.012[-4.590;6.613]	88.9%
Age									
<60	5	1.826[1.010; 3.304]	78.0%	34	1.750[0.610; 2.890]	100%	15	0.589[0.224;0.953]	93.2%
≥60	17	2.034[1.638; 2.525]	81.0%	14	1.700[-0.79; 3.478]	99.0%	–	–	–
Sample size									
<310	5	2.209[1.357;3.596]	35.1%	32	1.259[0.908; 1.610]	95.0%	12	0.788[0.325;1.252]	93.2%
≥310	17	1.844[1.482; 2.295]	79.0%	16	2.671[-0.166; 5.508]	100.0%	5	0.331[0.059;0.603]	83.7%
HbA1c(%)									
<8%	3	1.818[1.219;2.711]	0.0%	24	0.978[0.680;1.275]	94.9%	10	0.620[0.291;0.949]	84.4%
≥8%	6	1.742[12.470; 2.433]	40%	10	0.891[0.364;1.419]	93.3%	1	0.242[-0.068;0.551]	–
Albuminuria									
Microalbuminuria	–	–	–	16	0.671[0.474; 0.868]	86.0%	4	0.570[0.215;0.923]	44.0%
Macroalbuminuria	–	–	–	11	1.603[1.057; 2.149]	95.0%	4	1.021[0.466;1.577]	74.4%
eGFR(mL/min/1.73 m^2^)									
≥90	6	2.220[1.410; 3.494]	39.0%	18	1.951[0.641; 3.312]	100.%	2	0.338[-0.774;1.451]	0.00%
<90	9	1.916[1.442; 2.546]	40.0%	15	2.003[-0.694; 4.701]	100.0%	10	0.659[0.348;0.971]	86.5%
Disease duration(year)									
<10	8	1.693[1.160; 2.469]	84.0%	17	1.003[0.620;1.387]	94.4%	10	0.51[0.237;0.783]	86.0%
≥10	2	1.367[0.840;2.223]	0.0%	10	1.046[0.515;1.577]	96.7%	1	0.511[0.071;0.951]	

#### 3.2.2 DN incidence in high and low NLR groups

The meta-analysis covered 22 datasets from 13 studies (17, 26, 28, 36, 52, 54–56, 64, 67, 72–74) (**Figure 2B**). A random-effects model was adopted due to significant heterogeneity (I^2^ = 100%, *P*<0.01). It was found that the high NLR group had an incidence of DN 1.94 times higher than the low NLR group (OR=1.941, 95% CI: 1.609-2.341), suggesting a close association of high NLR with DN.

Subgroup analyses showed that the high heterogeneity in the pooled result might be attributed to variations in influencing factors like region, sample size, HbA1c, eGFR, and disease duration (**Table 1**). No statistically significant difference was observed among subgroups (**Table 1**).

#### 3.2.3 Differences in PLR levels between DN and non-DN patients

**Figure 3:**
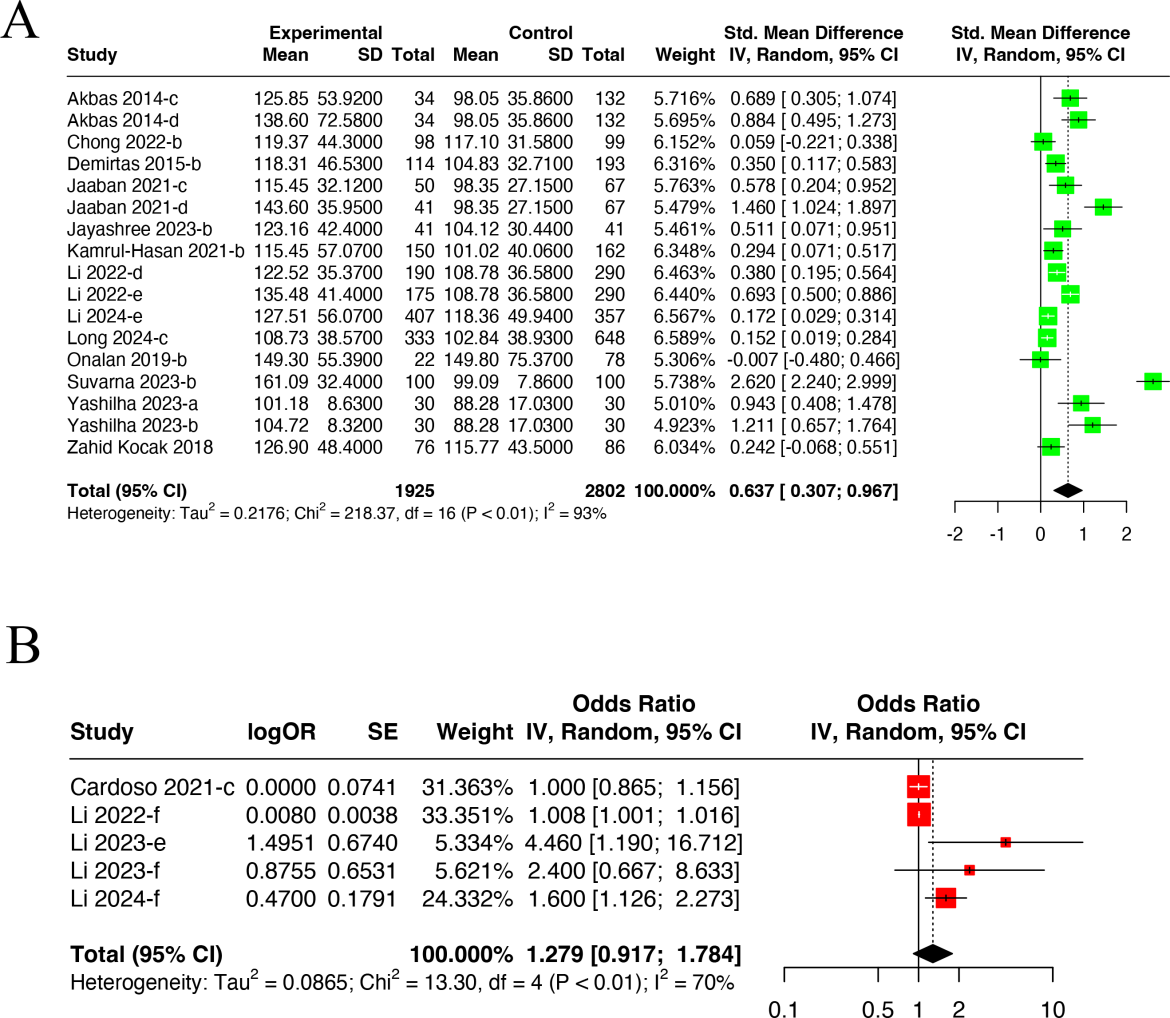
Forest plots illustrating the outcomes of the connection between PLR and DN incidence. **(A)** Forest plots for PLR levels in DN patients; **(B)** Forest plots for incidence of DN in high PLR and low PLR. PLR, Platelet-to-Lymphocyte Ratio.

Seventeen datasets from 13 studies (24, 32, 34, 44, 45, 47, 52, 54, 56, 58, 62, 69, 71) containing 1,925 DN patients and 2,802 non-DN patients (control group) were incorporated into the meta-analysis (**Figure 3A**). A random-effects model was adopted due to significant heterogeneity (I^2^ = 93.0%, P<0.001). DN patients had higher PLR levels than non-DN ones (SMD=0.637, 95% CI: 0.307-0.967).

Subgroup analyses revealed that the variation in UACR might contribute to considerable heterogeneity (**Table 1**). The predictive value of NLR as a continuous variable for DN vanished in subgroups with patients from America, HbA1c greater than 8%, and eGFR greater than 90 mL/min/1.73 m^2^, but it still had statistical significance in other subgroups.

#### 3.2.4 DN incidence in high and low PLR groups

Five datasets from four studies (28, 52, 54, 55) were incorporated into the meta-analysis (**Figure 3B**). A random-effects model was adopted due to significant heterogeneity (I^2^ = 70%, P<0.01). The DN incidence displayed no statistically significant difference between the high and low PLR groups (OR=1.279 1, 95% CI: 0.917-1.784).

#### 3.2.5 Differences in MLR levels between DN and non-DN patients

**Figure 4:**
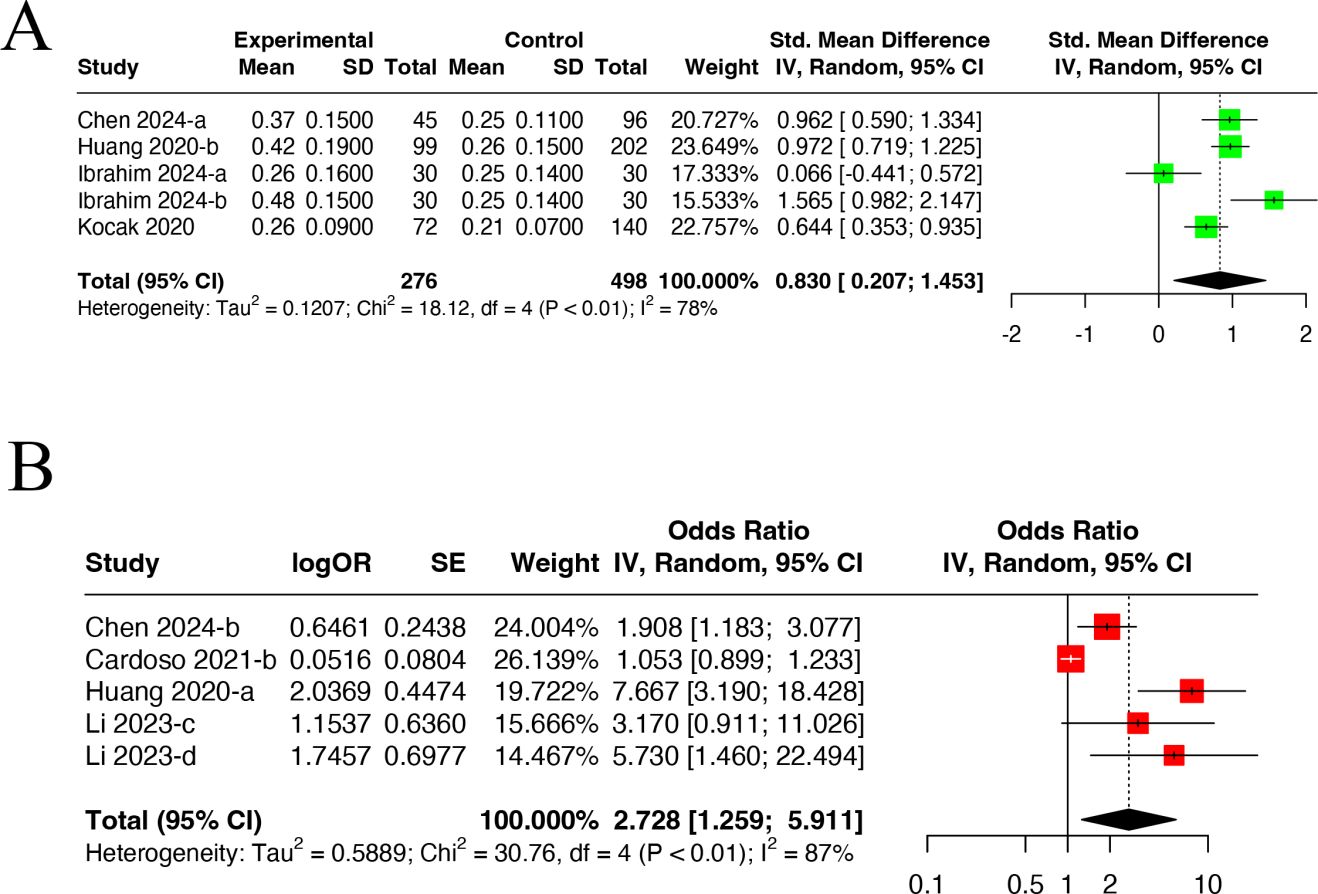
Forest plots illustrating the outcomes of the connection between MLR and DN incidence. **(A)** Forest plots for MLR levels in DN patients; **(B)** Forest plots for incidence of DN in high MLR and low MLR. MLR, Monocyte-to-Lymphocyte Ratio.

Five datasets from four studies (29, 41, 43, 71) containing 276 DN patients and 498 non-DN patients (control group) provided data for the meta-analysis (**Figure 4A**). A random-effects model was utilized due to high heterogeneity (I^2^ = 78.0%, P<0.01). DN patients had higher MLR levels than non-DN ones (SMD=0.830, 95% CI: 0.207-1.453).

#### 3.2.6 DN incidence in high and low MLR groups

Five datasets from four studies (28, 29, 41, 55) provided data for the meta-analysis (**Figure 4B**). A random-effects model was utilized due to high heterogeneity (I^2^ = 70%, P<0.01). It was found that the high MLR group had an incidence of DN 2.73 times higher than the low MLR group (OR=2.728, 95% CI: 1.259-5.911).

#### 3.2.7 Differences in SII levels between DN and non-DN patients

**Figure 5:**
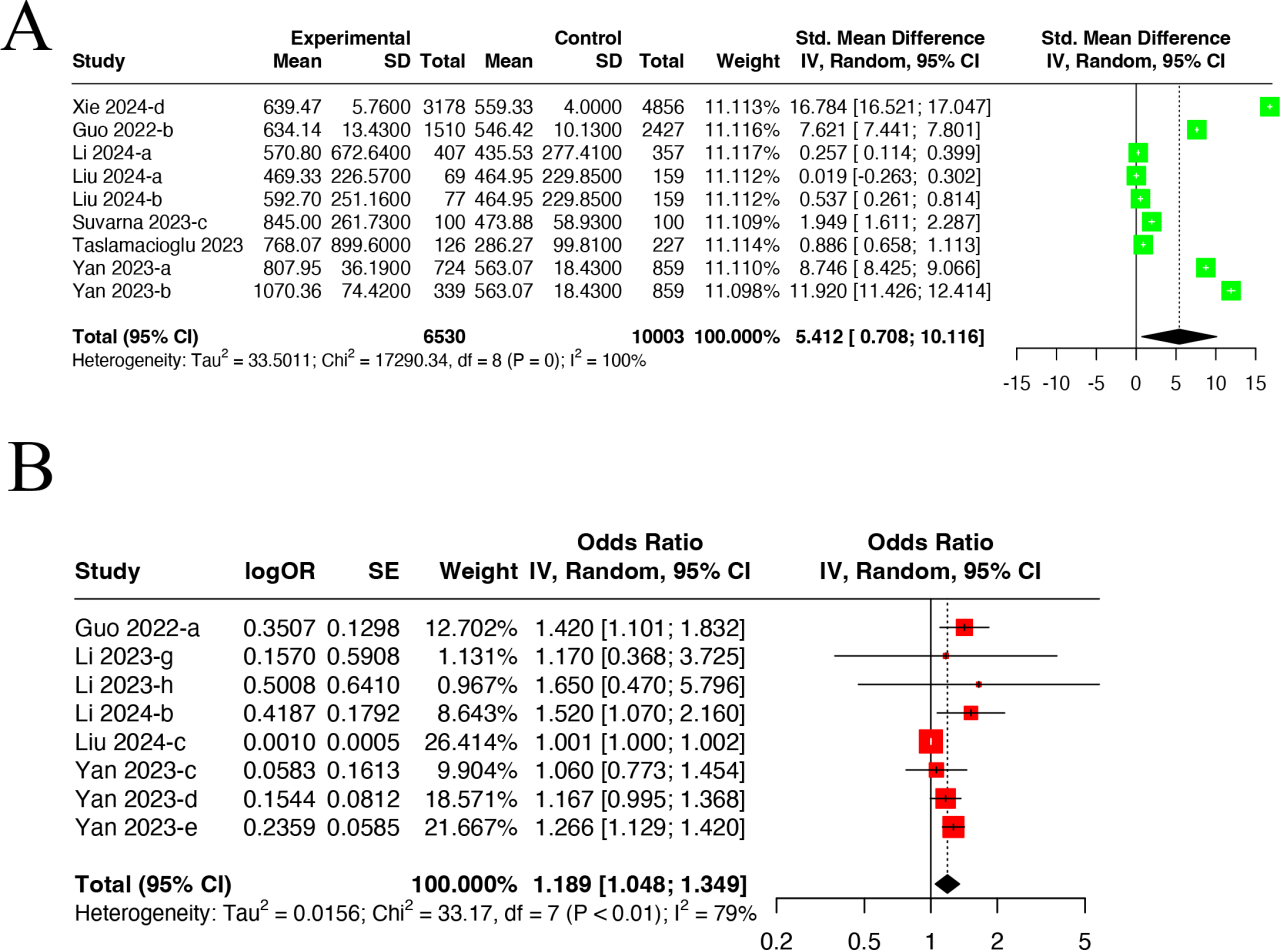
Forest plots illustrating the outcomes of the connection between SII and DN incidence. **(A)** Forest plots for SII levels in DN patients; **(B)** Forest plots for incidence of DN in high SII and low SII. SII, Systemic Immune-Inflammation Index.

The meta-analysis included nine datasets from seven studies (12, 22, 37, 52, 62, 63, 68) containing 6,530 DN patients and 10,003 non-DN patients (control group) (**Figure 5A**). A random-effects model was utilized due to high heterogeneity (I^2^ = 100.0%, P<0.001). DN patients had higher SII levels than non-DN ones (SMD=5.412, 95% CI: 0.708-10.116).

#### 3.2.8 DN incidence in high and low SII groups

The meta-analysis included nine datasets from five studies (12, 37, 52, 55, 68) (**Figure 5B**). A random-effects model was utilized due to high heterogeneity (I^2^ = 79%, P<0.01). It was found that the high SII group had an incidence of DN 1.19 times higher than the low SII group (OR=1.189, 95% CI: 1.048-1.349).

#### 3.2.9 DN incidence in high and low SIRI groups

The meta-analysis was conducted with three datasets from two studies (12, 55) (**Supplementary Figure S1**). A random-effects model was adopted. It was found that the high SIRI group had an incidence of DN 2.20 times higher than the low SIRI group (OR=2.197, 95% CI: 1.545-3.124). There was no heterogeneity (I^2^ = 0%, P=0.57).

### 3.3 Association of immune-inflammation indexes with DN prognosis: meta-analysis

Twelve datasets from eight studies (16, 22, 23, 28, 30, 59, 66, 72) containing 15,670 patients reported the relationship between high NLR and poor prognosis of DN (**Supplementary Figure S2**). Analysis of the pooled effect showed that the high NLR group was more prone to a poor prognosis than the low NLR group (OR: 1.372, 95% CI: 1.160-1.624, I^2^ = 83%).

Subgroup analyses were conducted based on different outcomes. Analysis of the pooled effect showed that the high NLR group had cardiovascular mortality and incidence of renal failure in DN 1.75 and 1.10 times, respectively, higher than the low NLR group (**Table 2**). However, all-cause mortality and eGFR decline had no statistically significant difference between the two groups. Besides, the variation in these outcomes might contribute to considerable heterogeneity.

**Table 2:** Subgroup analysis of the relationship between NLR with DN prognosis based on outcome.

Subgroup	Studies	Pooled OR (95% CI)	I^2^
All-cause mortality	Sato 2017, Xie 2024-a, Zeng 2024-a	1.377 [0.721; 2.628]	93%
Cardiovascular mortality	Xie 2024-b, Zeng 2024-b	1.748 [1.196; 2.555]	0%
eGFR decline	Akase 2020-a, Akase 2020-b, Wheelock-2018, Zhang 2022-e, Zhang 2022-f	1.496 [0.939; 2.385]	29%
Renal failure	Cheng 2020, Cardoso 2021	1.101 [1.018; 1.191]	0%

### 3.4 Sensitivity analyses

Leave-one-out sensitivity analyses were performed to examine the stability of the results (**Supplementary Figures S3**, **S4**). The pooled results of “Differences in MLR levels between DN and non-DN patients” became statistically no significant after the study “Chen 2024-a” “Huang 2020-b” and “Koack 2020” were removed (**Supplementary Figure S3E**). The pooled results of “ Differences in SII levels between DN and non-DN patients” became statistically no significant after the study “Guo 2022-b” “Survarna 2023-c” “Taslamacioglu 2023” “Yan 2023-a” and “Yan 2023-b” were removed (**Supplementary Figure S3G**). This may suggest a degree of uncertainty regarding the robustness of the pooled results for continuous variables in MLR and SII. However, the other results demonstrated stability, indicating that the meta-analysis results were robust despite significant heterogeneity among the included studies.

### 3.5 Publication bias

**Figure 6:**
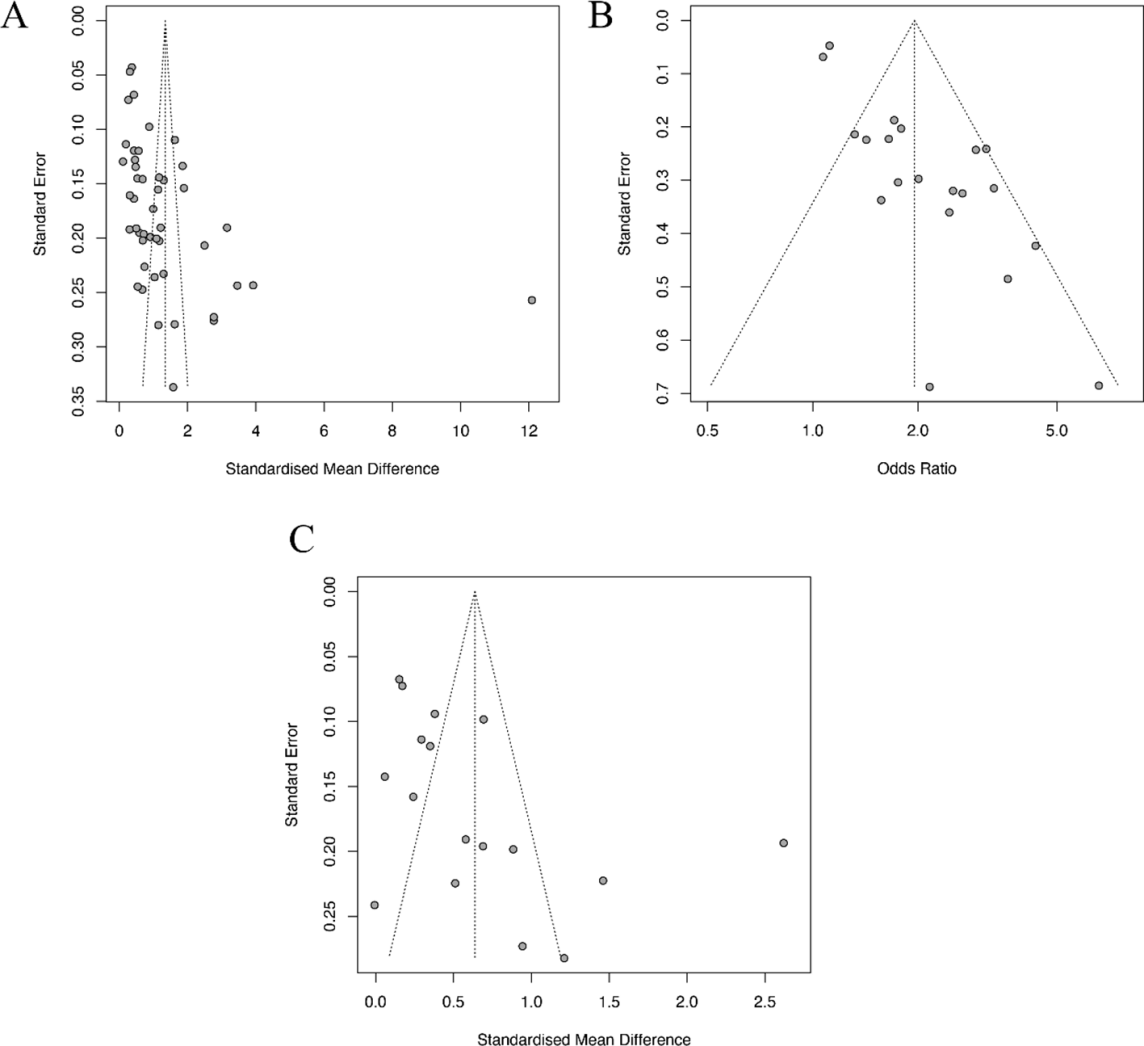
Funnel plot of publication bias between immune-inflammation index and DN incidence. **(A)** Funnel plot for NLR continues (3.2.1); **(B)** Funnel plot for NLR categorical (3.2.2); **(C)** Funnel plot for PLR continuous (3.2.3).

Funnel plots were used to evaluate publication bias in the combined results of more than 10 studies included (**Figure 6**; **Supplementary Figure S5**). The Egger’s test was further used to evaluate the asymmetry observed in the funnel plot. The results suggest that the following combined analysis may have publication bias: “3.2.1 Differences in NLR Levels between DN and non-DN Patients” (P=0.046), “3.2.2 Incidence of DN in High and low NLR Groups” (P<0.001), “3.2.3 Differences in PLR Levels between DN and non-DN Patients” (P=0.022), and “3.3 The correlation between immune inflammatory indicators and the prognosis of DN (P<0.001).

## 4 Discussion

Systemic inflammation is increasingly implicated in the pathogenesis and poor prognosis of DN (75). Hematological studies in T2DM patients show elevated leukocy (76, 77), indicating an active inflammatory response that may drive disease progression. Given the limitations of traditional markers like serum creatinine and proteinuria, novel indicators are needed. Immune-inflammation indexes, including NLR, PLR, MLR, SII, and SIRI, provide sensitive assessments of systemic inflammation (11). This study is the first large-scale analysis (56 studies, 53,278 participants) to examine their roles in DN. Notably, elevated NLR was associated with increased risks of adverse outcomes, including cardiovascular mortality and renal failure progression, highlighting its potential as a prognostic biomarker for DN. This study indicates that elevated immune-inflammatory indices are associated with the development and progression of DN, thereby offering clinicians a novel means to aid in the prevention of DN onset and the monitoring of its progression.

Liu et al. (2018) reported the expression changes of NLR in DN and found that NLR was significantly elevated in patients with DN (SMD = 0.63) (2). Consistent with these previous findings, our study demonstrated that the incidence of DN in the high NLR group was 1.94 times that of the low NLR group, and that NLR was significantly increased in DN patients (SMD = 1.73). Building upon Liu’s foundational work, our study leveraged the most up-to-date data, with a broader search scope, a larger number of included studies, and a substantially greater sample size. Moreover, our research not only assessed NLR but also incorporated several emerging immune-inflammatory markers such as PLR, MLR, SII, and SIRI, providing a comprehensive and systematic analysis of their associations with DN risk. Notably, we were the first to quantitatively analyze the relationship between high NLR and DN prognosis across multiple studies. Our conclusions not only reinforce the predictive value of NLR for the occurrence of DN but also systematically summarize its prognostic significance in DN. Compared to previous studies, our research offers a more comprehensive perspective and greater clinical relevance. Besides, the sensitivity analyses demonstrated the stability of our results.

Moreover, subgroup analyses were conducted to identify the source of heterogeneity (78, 79). First, the significant heterogeneity in the pooled results of NLR as a categorical variable could be attributed to the combination of several confounders. Specifically, inter-study geographic differences, diversity of HbA1c levels, sample size, inconsistency in eGFR, and variability in disease duration could explain the heterogeneity in DN incidence in the high NLR group. However, the heterogeneity in the results of NLR as a continuous variable was not adequately explained by the subgroup analyses. The heterogeneity in the results of PLR as a continuous variable was possibly related to proteinuria. Notably, subgroup analyses revealed higher pooled effect sizes for PLR in patients with macroalbuminuria than those with microalbuminuria, suggesting a potential association between immune-inflammation indexes and renal function in DN patients.

This study focused on NLR’s association with DN prognosis. As reported previously, NLR is associated with adverse outcomes of various diseases (cardiovascular disease (80), T2DM (81), coronary artery disease (6), malignancies (14, 82), and sepsis (83)), with enhanced chronic inflammation and elevated NLR considered as its pathogenesis (6, 84). However, the association of NLR with DN and its prognosis is poorly understood. To our knowledge, this meta-analysis filled the research gap by analyzing the association of high NLR with the adverse prognosis of DN for the first time. The results of the meta-analysis showed that there was a certain correlation between high NLR and poor prognosis in patients with DN, consistent with previous studies (28, 30, 59, 85, 86). However, subgroup analyses indicated that NLR demonstrated potential predictive value for cardiovascular mortality and renal failure progression in patients with DN. Nevertheless, no statistically significant associations were observed with all-cause mortality or eGFR decline. This discrepancy may stem from the limited number of included studies, substantial sample heterogeneity, and a paucity of high-quality prospective investigations, collectively compromising the robustness and statistical power of these specific findings. Based on the current evidence, NLR shows promise for predicting adverse outcomes in the DN population. However, before NLR becomes an effective prognostic prediction tool, more cohort tracking data support is still needed. Future research should prioritize large-scale, multicenter longitudinal studies to definitively establish clinical thresholds for various immune-inflammatory biomarkers and validate their practical utility in prognostic assessment for DN.

An accumulating body of research has recently indicated the key role of inflammatory responses in DN development (8, 44). Neutrophils are important elements in the inflammatory response, and they can be activated by metabolic disorders such as hyperglycemia. Then activated neutrophils can release such inflammatory mediators as IL-1, TNF-α, chemokines, and ROS, which can further worsen the inflammatory response and injury in renal tissues (87). Monocytes can also be activated upon stimulation with inflammatory factors to release inflammatory mediators and participate in fibrosis, worsening the inflammation and injury of renal tissues and thus facilitating DN progression (87, 88). In addition, activated lymphocytes may be implicated in the fibrosis of renal tissues by releasing growth factors and cytokines, thus promoting glomerulosclerosis and interstitial fibrosis and aggravating the pathological changes of DN (89, 90). Activated platelets in DN can release growth factors and pro-fibrotic factors and interact with endothelial cells to facilitate endothelial cell injury and fibrosis, leading to glomerulosclerosis and interstitial fibrosis as well as vascular endothelial dysfunction, worsening renal microcirculatory disorders, and tissue hypoxia, ultimately promoting DN development. Meanwhile, abnormally activated platelets may exacerbate vascular injury and microcirculatory disorders, causing renal ischemia and reperfusion injury, and further aggravating kidney injury (89, 91).

Different immune-inflammation indexes correspond to different inflammation statuses in DN. Specifically, elevated NLR in DN suggests enhanced inflammation and immune cell activity, increased release of inflammatory mediators, and inflammation-related injury. Elevated PLR implies more active inflammatory responses in DN and may also correlate with increased platelet activation. Then platelet activation and aggregation may lead to thrombosis (92). Abnormally elevated SII suggests systemic inflammation and increases in the systemic pain index and inflammatory markers (CRP, WBC, and NLR), while high levels of inflammatory markers can affect the vascular endothelial cell function and increase oxidative stress and fibrosis, thus damaging the structure and function of glomerular filtration membrane, and ultimately facilitating DN development. These immune-inflammation indexes with the above characteristics provide important clues for knowing the inflammation status in DN, which can help physicians develop more effective treatment strategies and monitor disease progression. A meta-analysis has shown that anti-inflammatory therapy can effectively lower the risk of cardiovascular events in T2DM patients, suggesting that targeting inflammation can reduce the risk of diabetic complications (93). Future studies are required to further identify whether DN patients with elevated NLR or other inflammation indexes can benefit from anti-inflammatory therapies and interventions, thereby ameliorating their quality of life and prognosis.

However, this meta-analysis still had some limitations worth considering. First, all of the eligible data originated from Asia and the Americas, especially China, Turkey, the United States, and India. Therefore, the conclusions should be interpreted in this geographic context and generalized with caution to Europe, Africa, and other regions. In addition, even after subgroup analyses, some of the pooled results (e.g., the pooled results of NLR as a continuous variable) still had heterogeneity that could not be fully explained. Although it was difficult to identify the source of heterogeneity, it was hypothesized that race, treatment, and other factors possibly had a potential impact on the heterogeneity in the included studies. Notably, the inability to standardize cut-off values may be a source of heterogeneity. This is primarily because the cut-off values varied greatly across studies, and a considerable proportion of the reported ORs were derived from multivariate analyses, which did not provide information on the specific cut-off values used (37, 41). Finally, it was confirmed by funnel plots and Egger’s tests that the pooled results were affected by publication bias, and therefore the conclusions of this meta-analysis should be interpreted with the potential impact of publication bias considered.

## 5 Conclusion

In conclusion, the association of blood-derived immune-inflammation indexes with the incidence and prognosis of DN was comprehensively assessed in this meta-analysis. High-level immune-inflammation indexes may serve as predictors for DN incidence, and high NLR is potentially associated with the occurrence of poor prognosis of DN. In the future, more longitudinal studies are needed to clarify the association between immune-inflammation indexes and DN prognosis. This study offers realistic support to the role of systemic inflammation in DN onset and progression and reveals the significant potential of immune-inflammation indexes as biomarkers of inflammation for assessing the risk and prognosis of DN.

## Supplementary material

Supplementary Figure 1 Forest plots showing the outcomes for incidence of DN in high SIRI and low SIRI. SIRI, Systemic Inflammation Response Index.

Supplementary Figure 2 Forest plots showing the outcomes for prognosis of DN in high NLR and low NLR.

Supplementary Figure 3 The sensitive analysis of selected studies. (A) NLR levels in DN patients;(B) incidence of DN in high NLR and low NLR;(C) PLR levels in DN patients;(D) incidence of DN in high PLR and low PLR;(E) MLR levels in DN patients; (F) incidence of DN in high MLR and low MLR;(G) SII levels in DN patients;(H) incidence of DN in high SII and low SII;(I) incidence of DN in high SIRI and low SIRI

Supplementary Figure 4 The sensitive analysis of the prognosis of DN in high NLR and low NLR.

Supplementary Figure 5 Funnel plot of publication bias between NLR and DN prognosis.
