# Supplementary material for: Association of immune-inflammation indexes with incidence and prognosis of diabetic nephropathy: a systematic review and meta-analysis
Source: Front Endocrinol (Lausanne). 2025 Aug 18;16:1532682. doi: 10.3389/fendo.2025.1532682 (PMC12399399; doi:10.3389/fendo.2025.1532682)
Supplement: Supplementary file 1 [file Table1.docx]

**Table S1.** Full search strategy.

Pubmed-396

((((Systemic inflammation response index) OR (SIRI)) OR ((Systemic immune inflammation index) OR (SII))) OR ((Ratio) AND ((((("Lymphocytes"[Mesh]) OR ((Lymphocyte) OR (Lymphoid Cell*))) OR (("Monocytes"[Mesh]) OR (Monocyte))) OR ((((((Neutrophil) OR (Polymorphonuclear Neutrophil*)) OR (Polymorphonuclear Leukocyte*)) OR (LE Cell*)) OR (Neutrophil Band Cell*)) OR ("Neutrophils"[Mesh]))) OR ((((Blood Platelet) OR (Platelet*)) OR (Thrombocyte*)) OR ("Blood Platelets"[Mesh]))))) AND (((((Diabetic Nephropathy) OR (Nephropathies, Diabetic)) OR (Diabetic Kidney Disease)) OR (Diabetic Glomerulosclerosis)) OR ("Diabetic Nephropathies"[Mesh]))

Embase-859


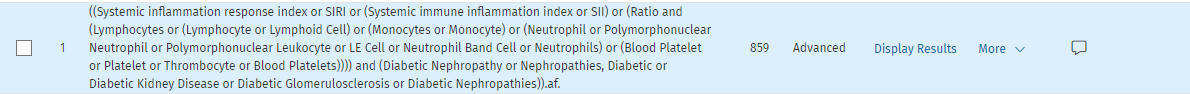


Cochrane-65


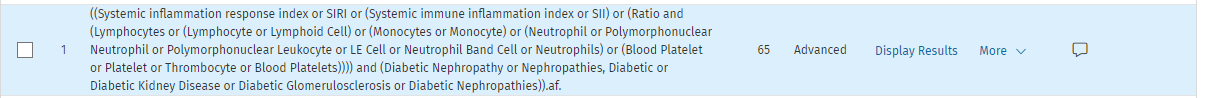


WOS-697

((((Systemic inflammation response index) OR (SIRI)) OR ((Systemic immune inflammation index) OR (SII))) OR ((Ratio) AND (((((Lymphocytes) OR ((Lymphocyte) OR (Lymphoid Cell))) OR ((Monocytes) OR (Monocyte))) OR ((((((Neutrophil) OR (Polymorphonuclear Neutrophil)) OR (Polymorphonuclear Leukocyte)) OR (LE Cell)) OR (Neutrophil Band Cell)) OR (Neutrophils))) OR ((((Blood Platelet) OR (Platelet)) OR (Thrombocyte)) OR (Blood Platelets))))) AND (((((Diabetic Nephropathy) OR (Nephropathies, Diabetic)) OR (Diabetic Kidney Disease)) OR (Diabetic Glomerulosclerosis)) OR (Diabetic Nephropathies)) (Topic)

**Table S2.** Study characteristics.

| Author and year | Study period | Region | Study design | Sample size | Gender, M/F | Age, years ± SD | HbA1c, mmol/mol OR % ± SD | eGFR mL/min/1.73 m2 ± SD | Diabetes duration, years ± SD | Included immune- inflammation types |
| --- | --- | --- | --- | --- | --- | --- | --- | --- | --- | --- |
| Akbas 2014[1] | - | Turkey | case-control | 200 | 103/97 | 57.28±10.64 | 8.64±2.67 | 94.77±34.78 | 7.25±6.16 | NLR, PLR |
| Akase 2020[2] | 2017-2019 | Japan | cohort | 358 | 184/174 | 74.66±10.39 | 6.68±0.76 | - | 11.77±8.96 | NLR |
| Assulyn 2020[3] | 2014-2017 | Israel | case-control | 168 | 84/84 | 65.02±10.56 | 7.80±1.56 | - | 13.08±7.35 | NLR |
| Bhattacharyya 2021[4] | 2017-2019 | India | case-control | 80 | 47/33 | - | - | - | - | NLR |
| Bloch 2020[5] | 2018-2020 | Pakistan | case-control | 132 | 68/64 | 51.95±11.22 | - | - | - | NLR |
| Chen 2024[6] | - | China | case-control | 141 | 69/72 | 57.89±13.35 | 9.53±3.61 | 108.81±38.33 | - | MLR |
| Cheng 2020[7] | 2012-2017 | China | cohort | 921 | 494/427 | 54.69±11.63 | - | 56.36±63.31 | - | NLR |
| Chollangi 2023[8] | 2020-2022 | India | case-control | 90 | 53/37 | 62.25±9.70 | 8.55±1.48 | - | - | NLR |
| Chong 2022[9] | 2019-2021 | China | case-control | 197 | 0/197 | 30.76±4.73 | - | 197.86±36.84 | 30.76±4.73 | NLR,PLR |
| Ciray 2015[10] | 2013-2014 | Turkey | case-control | 114 | 41/73 | 59.7±11.3 | 9.15±2.34 | 77.51±27.60 | 9.1±6.5 | NLR |
| Cardoso 2021[11] | 2004-2019 | Brazil | cohort | 697 | 270/427 | 60.0±9.6 | - | - | 8.70±8.91 | NLR,PLR,MLR |
| Demirtas 2015[12] | - | Turkey | case-control | 307 | 124/183 | 51.65±9.68 | 8.64±2.61 | - | - | NLR |
| Fang 2024[13] | 2021-2022 | China | case-control | 90 | 47/43 | 64.06±8.70 | 7.95±1.48 | 80.69±21.08 | 14.10±10.19 | NLR |
| Gao 2024[14] | 2021 | China | case-control | 1040 | 490/550 | 71.9±5.5 | - | 80.2±15.7 | - | NLR |
| Guo 2022[15] | 2011-2018 | US | case-control | 3937 | 2040/1897 | 59.19±3.98 | - | 83.81±11.70 | - | NLR,SII |
| Gupta 2018[16] | 2018 | Inida | case-control | 300 | 162/138 | 48.08±26.90 | 7.66±0.96 | 103.66±33.07 | 10.63±5.66 | NLR |
| Gurmu 2022[17] | 2019-2020 | ethiopia | case-control | 199 | 88/111 | 55.32±11.85 | - | - | - | NLR |
| Huang 2015[18] | 2013-2014 | China | case-control | 253 | 113/140 | 50.32±10.24 | - | - | - | NLR |
| Huang 2017[19] | 2014-2015 | China | case-control | 321 | 176/145 | 56.55±10.69 | 9.10±2.07 | - | - | NLR |
| Huang 2020[20] | 2015-2016 | China | case-control | 301 | 193/108 | 59.06±9.29 | 7.27±2.04 | 100.81±16.99 | 7.15±3.28 | MLR |
| Ibrahim 2024[21] | 2020-2023 | Egypt | case-control | 90 | 39/51 | 50.25±7.82 | 9.41±2.07 | 71.23±36.64 | - | MLR |
| Jaaban 2021[22] | 2017-2019 | Syria | case-control | 158 | 92/66 | 57.08±8.65 | 8.39±1.44 | 91.46±19.66 | 8.39±1.44 | NLR,PLR |
| Jayashree 2023[23] | 2020-2022 | India | case-control | 82 | - | 54.37±9.06 | - | 75.49±58.12 | 10.01±6.26 | NLR,PLR |
| Kahraman 2016[24] | 2013-2014 | Turkey | case-control | 112 | 39/73 | 60.51±9.63 | 9.18±2.53 | 112.44±82.80 | 5.27±3.46 | NLR |
| Kamrul-Hasan 2021[25] | 2018-2019 | Bangladesh | case-control | 312 | 141/171 | 50.19±10.88 | 8.78±2.04 | 82.82±22.36 | 6.83±6.11 | NLR,PLR |
| Kawamoto 2019[26] | 2017 | Japan | case-control | 386 | 199/187 | 74.82±10.92 | 6.70±0.83 | - | - | NLR |
| Kaya 2019[27] | 2014 | Turkey | case-control | 355 | - | 57.83±9.71 | - | - | - | NLR |
| Khandare 2017[28] | 2015-2016 | India | case-control | 115 | 51/64 | 51.14±11.37 | 8.13±1.42 | 91.09±28.35 | - | NLR |
| Kocak 2020[29] | 2017-2018 | Turkey | case-control | 212 | 95/117 | 59.90±8.54 | - | - | 7.77±4.57 | MLR |
| Li 2022[30] | 2018-2021 | China | case-control | 655 | 351/304 | 59.94±10.63 | - | 93.82±14.68 | 9.25±10.49 | NLR,PLR |
| Li 2023[31] | 1999-2020 | US | case-control | 7153 | 3194/3959 | - | - | 90.55±29.84 | - | NLR,MLR,PLR,SII,SIRI |
| Li 2024[32] | 2018-2020 | China | case- control | 203 | 133/70 | 59.04±10.87 | 8.75±2.14 | 75.96±43.00 | - | NLR |
| Li 2024[33] | 2020-2023 | China | case-control | 1058 | 721/337 | 54.67±12.86 | 8.59±2.20 | 102.01±23.84 | 9.32±7.10 | NLR,PLR,SII |
| Liu 2024[34] | 2020-2021 | China | case-control | 303 | 202/101 | 59.88±10.03 | 9.01±2.24 | 86.81±24.79 | 10.98±7.58 | SII,SIRI |
| Long 2024[35] | 2021-2023 | China | case-control | 981 | 522/459 | 59.00±10.39 | - | - | 8±5.94 | NLR,PLR |
| Mattared 2019[36] | 2017 | Egypt | case-control | 60 | - | 59.75±9.15 | 7.1±1.00 | 96.25±11.43 | - | NLR |
| Onalan 2019[37] | 2018 | Turkey | case-control | 100 | 48/52 | 56.34±12.55 | 9.62±2.24 | - | - | NLR,PLR |
| Rakesh 2024[38] | 2018-2019 | India | case-control | 104 | 76/28 | 53.3±11.8 | - | 86.75 | 6.2±5.92 | NLR |
| Sato 2017[39] | 2009-2014 | Japan | cohort | 78 | 51/27 | 63.4±11.7 | 5.9±0.7 | 4.9±2.3 | - | NLR |
| Singh 2022[40] | 2019-2020 | India | case-control | 324 | 195/129 | 56.07±10.45 | 8.16±2.42 | 70.16±38.44 | 13.06±3.72 | NLR |
| Subramani 2023[41] | 2021-2020 | India | case-control | 160 | 92/68 | 57.13±8.63 | 8.40±1.44 | 91.20±19.70 | 9.26±2.84 | NLR |
| Suvarna 2023[42] | 2021 | India | case-control | 200 | 129/71 | 57.5±11.26 | - | - | - | NLR,PLR,SII |
| Taslamacioglu 2023[43] | 2020-2022 | Turkey | case-control | 353 | 148/205 | 54.36±9.62 | - | 106.34±24.68 | - | SII |
| TuTan 2023[44] | 2021-2023 | Turkey | case-control | 327 | 148/179 | 60.83±11.93 | 11.34±1.42 | - | - | NLR |
| Wang 2021[45] | 2018-2020 | China | case-control | 2045 | 1188/857 | 61.30±10.84 | 9.05±2.35 | 82.31±30.97 | - | NLR |
| Wheelock[46] | 2002-2012 | France | cohort | 941 | 397/544 | 64.16±10.6 | 7.9±1.6 | 76±21 | 12.02±2.18 | NLR |
| Xi 2021[47] | 2019-2020 | China | case-control | 1095 | 472/623 | - | - | - | - | NLR |
| Xie 2024[48] | 2005-2020 | US | cohort | 8034 | 4148/3886 | 59.81±3.81 | - | 83.21±10.56 | - | NLR,SII |
| Yan 2023[49] | 2012-2015 | China | case-control | 1922 | 975/947 | 60.72±11.29 | 9.30±2.51 | 87.37±29.55 | 8.12±6.49 | SII |
| Yashilha 2023[50] | 2018-2022 | India | cohort | 90 | 35/55 | - | 8.78±1.21 | 93.01±16.19 | - | PLR |
| Yay 2024[51] | 2022-2023 | Turkey | case-control | 164 | 71/93 | 57.29±12.48 | 7.64±1.77 | 85.67±25.72 | - | NLR |
| Zahid Kocak 2018[52] | 2017 | Turkey | case-control | 162 | 83/79 | 58.90±9.58 | 7.88±1.61 | - | 7.35±7.21 | PLR |
| Zhang 2022[53] | 2017-2019 | China | Case-control | 1192 | 659/533 | 61.47±5.19 | 8.96±0.11 | 91.47±26.83 | - | NLR |
|  | 2016-2019 | China | cohort | 2060 | 949/1111 | 68.93±0.13 | 7.60±0.29 | 101.41±0.56 | - | NLR |
| Zeng 2024[54] | 1999-2018 | US | cohort | 2581 | 1372/1209 | 63.80±0.36 | 7.38±0.05 | 69.81±0.78 | - | NLR |
| Zhang 2019[55] | 2009-2017 | China | case-control | 247 | 171/76 | 51.70±8.95 | 7.12±1.45 | 61.39±34.23 | 7±5.97 | NLR |
| Zhang 2019[56] | 2018 | China | case-control | 287 | 162/125 | 55.54±14.36 | 9.09±2.37 | 117.61±45.71 | 7.26±7.94 | NLR |

1. Akbas, E.M., et al., Association of epicardial adipose tissue, neutrophil-to-lymphocyte ratio and platelet-to-lymphocyte ratio with diabetic nephropathy. Int J Clin Exp Med, 2014. 7(7): p. 1794-801.

2. Akase, T., et al., Neutrophil-to-lymphocyte ratio is a predictor of renal dysfunction in Japanese patients with type 2 diabetes. Diabetes Metab Syndr, 2020. 14(4): p. 481-487.

3. Assulyn, T., et al., Neutrophil-to-lymphocyte ratio and red blood cell distribution width as predictors of microalbuminuria in type 2 diabetes. J Clin Lab Anal, 2020. 34(7): p. e23259.

4. Bhattacharyya, S., et al., A Cross-sectional Study to Assess Neutrophil Lymphocyte Ratio as a Predictor of Microvascular Complications in Type 2 Diabetes Mellitus Patients. Journal of Clinical and Diagnostic Research, 2021. 15(8): p. OC59-OC62.

5. Bloch, M.H., et al., Role of neutrophil / lymphocyte ratio in diabetes 2 nephropathy. Medical Forum Monthly, 2020. 31(7): p. 29-32.

6. Chen, J., et al., Low Levels of Metrnl are Linked to the Deterioration of Diabetic Kidney Disease. Diabetes Metabolic Syndrome and Obesity, 2024. 17: p. 959-967.

7. Cheng, Y., et al., Development and validation of a predictive model for the progression of diabetic kidney disease to kidney failure. Renal Failure, 2020. 42(1): p. 550-559.

8. Chollangi, S., et al., Exploring the Correlates of Hematological Parameters With Early Diabetic Nephropathy in Type 2 Diabetes Mellitus. Cureus, 2023. 15(5): p. e39778.

9. Chong, H., et al., The diagnostic model for early detection of gestational diabetes mellitus and gestational diabetic nephropathy. Journal of Clinical Laboratory Analysis, 2022. 36(9).

10. Ciray, H., et al., Nephropathy, but not Angiographically Proven Retinopathy, is Associated with Neutrophil to Lymphocyte Ratio in Patients with Type 2 Diabetes. Exp Clin Endocrinol Diabetes, 2015. 123(5): p. 267-71.

11. Cardoso, C.R.L., N.C. Leite, and G.F. Salles, Importance of hematological parameters for micro- and macrovascular outcomes in patients with type 2 diabetes: the Rio de Janeiro type 2 diabetes cohort study. Cardiovasc Diabetol, 2021. 20(1): p. 133.

12. Demirtas, L., et al., Association of hematological indicies with diabetes, impaired glucose regulation and microvascular complications of diabetes. Int J Clin Exp Med, 2015. 8(7): p. 11420-7.

13. Fang, Y., et al., Exploring the relations of NLR, hsCRP and MCP-1 with type 2 diabetic kidney disease: a cross-sectional study. Sci Rep, 2024. 14(1): p. 3211.

14. Gao, J.-L., et al., Neutrophil-to-lymphocyte ratio associated with renal function in type 2 diabetic patients. World Journal of Clinical Cases, 2024. 12(14).

15. Guo, W., et al., Systemic immune-inflammation index is associated with diabetic kidney disease in Type 2 diabetes mellitus patients: Evidence from NHANES 2011-2018. Front Endocrinol (Lausanne), 2022. 13: p. 1071465.

16. Gupta, N., et al., The relationship between neutrophil/lymphocyte ratio, albuminuria and renal dysfunction in diabetic nephropathy. Journal, Indian Academy of Clinical Medicine, 2018. 19(4): p. 265-268.

17. Gurmu, M.Z., et al., Neutrophil-lymphocyte ratio as an inflammatory biomarker of diabetic nephropathy among type 2 diabetes mellitus patients: A comparative cross-sectional study. SAGE Open Med, 2022. 10: p. 20503121221140231.

18. Huang, W., et al., Neutrophil-lymphocyte ratio is a reliable predictive marker for early-stage diabetic nephropathy. Clin Endocrinol (Oxf), 2015. 82(2): p. 229-33.

19. Huang, L., et al., Neutrophil-to-lymphocyte ratio in diabetic microangiopathy. International Journal of Clinical and Experimental Pathology, 2017. 10(2): p. 1223-1232.

20. Huang, Q., et al., Monocyte-lymphocyte ratio is a valuable predictor for diabetic nephropathy in patients with type 2 diabetes. Medicine (Baltimore), 2020. 99(19): p. e20190.

21. Ibrahim, H.M.M., et al., Monocyte lymphocyte ratio, IL 6, and their association with increased carotid intima-media thickness as simple predictive markers for nephropathy in Egyptian diabetic patients. Egyptian Journal of Internal Medicine, 2024. 36(1).

22. Jaaban, M., et al., Neutrophil-lymphocyte ratio and platelet-lymphocyte ratio as novel risk markers for diabetic nephropathy in patients with type 2 diabetes. Heliyon, 2021. 7(7): p. e07564.

23. Jayashree, K., et al., Circulating 18-Glycosyl Hydrolase Protein Chitiotriosidase-1 is Associated with Renal Dysfunction and Systemic Inflammation in Diabetic Kidney Disease. Int J Appl Basic Med Res, 2023. 13(3): p. 159-167.

24. Kahraman, C., et al., The relationship between neutrophil-to-lymphocyte ratio and albuminuria in type 2 diabetic patients: a pilot study. Arch Med Sci, 2016. 12(3): p. 571-5.

25. Kamrul-Hasan, A.B.M., et al., Evaluation of neutrophil-lymphocyte ratio and platelet-lymphocyte ratio as markers of diabetic kidney disease in Bangladeshi patients with type 2 diabetes mellitus. Journal of Diabetology, 2021. 12(1): p. 58-62.

26. Kawamoto, R., et al., Association of neutrophil-to-lymphocyte ratio with early renal dysfunction and albuminuria among diabetic patients. Int Urol Nephrol, 2019. 51(3): p. 483-490.

27. Kaya, Y., A. Karatas, and I. Irende, Relationship between glomerular filtration rate with uric acid and neutrophil to lymphocyte ratioin diabetic patients. Annals of Clinical and Analytical Medicine, 2019. 10(4): p. 436-440.

28. Khandare, S.A., et al., Study of Neutrophil-lymphocyte Ratio as Novel Marker for Diabetic Nephropathy in Type 2 Diabetes. Indian J Endocrinol Metab, 2017. 21(3): p. 387-392.

29. Kocak, M.Z., et al., Monocyte lymphocyte ratio As a predictor of Diabetic Kidney Injury in type 2 Diabetes mellitus; The MADKID Study. Journal of Diabetes and Metabolic Disorders, 2020. 19(2): p. 997-1002.

30. Li, L., Q. Shen, and S. Rao, Association of Neutrophil-to-Lymphocyte Ratio and Platelet-to-Lymphocyte Ratio with Diabetic Kidney Disease in Chinese Patients with Type 2 Diabetes: A Cross-Sectional Study. Ther Clin Risk Manag, 2022. 18: p. 1157-1166.

31. Li, X., et al., Association between neutrophil-to-lymphocyte ratio and diabetic kidney disease in type 2 diabetes mellitus patients: a cross-sectional study. Front Endocrinol (Lausanne), 2023. 14: p. 1285509.

32. Li, J.J., et al., Evaluating new biomarkers for diabetic nephropathy: Role of alpha2- macroglobulin, podocalyxin, alpha-L-fucosidase, retinol-binding protein- 4, and cystatin C. World Journal of Diabetes, 2024. 15(6): p. 1212-1225.

33. Li, J., et al., Association of the systemic immuno-inflammation index, neutrophil-to-lymphocyte ratio, and platelet-to-lymphocyte ratio with diabetic microvascular complications. Front Endocrinol (Lausanne), 2024. 15: p. 1367376.

34. Liu, W., S. Zheng, and X. Du, Association of Systemic Immune-Inflammation Index and Systemic Inflammation Response Index with Diabetic Kidney Disease in Patients with Type 2 Diabetes Mellitus. Diabetes Metab Syndr Obes, 2024. 17: p. 517-531.

35. Long, W., et al., Development of a predictive model for the risk of microalbuminuria: comparison of 2 machine learning algorithms. Journal of Diabetes and Metabolic Disorders, 2024.

36. Mattared, A.M., et al., Mean platelet volume and neutrophil to lymphocyte ratio in prediction of early diabetic nephropathy in type 2 diabetics. Diabetes Metab Syndr, 2019. 13(2): p. 1469-1473.

37. Onalan, E., N. Gozel, and E. Donder, Can hematological parameters in type 2 diabetes predict microvascular complication development? Pak J Med Sci, 2019. 35(6): p. 1511-1515.

38. Rakesh, B., N. Pradeep, and G. Nischal, Correlation between Neutrophil to Lymphocyte Ratio and Urine Albumin to Creatinine Ratio in Diabetic Nephropathy Patients: A Cross-sectional Study. Journal of Clinical and Diagnostic Research, 2024. 18(5): p. OC33-OC37.

39. Sato, H., et al., Pre-Dialysis Neutrophil-Lymphocyte Ratio, a Novel and Strong Short-Term Predictor of All-Cause Mortality in Patients With Diabetic Nephropathy: Results From a Single-Center Study. Ther Apher Dial, 2017. 21(4): p. 370-377.

40. Singh, A., et al., Neutrophil lymphocyte ratio: a reliable biomarker for diabetic nephropathy? International Journal of Diabetes in Developing Countries, 2022. 42(3): p. 523-528.

41. Subramani, M., et al., Role of neutrophil-lymphocyte ratio as a prognostic marker for type 2 diabetic nephropathy among Indians. Bioinformation, 2023. 19(4): p. 375-379.

42. Suvarna, R., et al., Association of clinical variables as a predictor marker in type 2 diabetes mellitus and diabetic complications. Biomedicine (India), 2023. 43(1): p. 335-340.

43. Taslamacioglu Duman, T., F.N. Ozkul, and B. Balci, Could Systemic Inflammatory Index Predict Diabetic Kidney Injury in Type 2 Diabetes Mellitus? Diagnostics (Basel), 2023. 13(12).

44. Tutan, D. and M. Doğan, Evaluation of Neutrophil/Lymphocyte Ratio, Low-Density Lipoprotein/Albumin Ratio, and Red Cell Distribution Width/Albumin Ratio in the Estimation of Proteinuria in Uncontrolled Diabetic Patients. Cureus, 2023. 15(8): p. e44497.

45. Wang, K., et al., Fibrinogen to Albumin Ratio as an Independent Risk Factor for Type 2 Diabetic Kidney Disease. Diabetes Metab Syndr Obes, 2021. 14: p. 4557-4567.

46. Wheelock, K.M., et al., White blood cell fractions correlate with lesions of diabetic kidney disease and predict loss of kidney function in Type 2 diabetes. Nephrol Dial Transplant, 2018. 33(6): p. 1001-1009.

47. Xi, C., et al., A Nomogram Model that Predicts the Risk of Diabetic Nephropathy in Type 2 Diabetes Mellitus Patients: A Retrospective Study. Int J Endocrinol, 2021. 2021: p. 6672444.

48. Xie, R., et al., Higher Circulating Neutrophil Counts Is Associated with Increased Risk of All-Cause Mortality and Cardiovascular Disease in Patients with Diabetic Kidney Disease. Biomedicines, 2024. 12(8).

49. Yan, P., et al., Association of systemic immune-inflammation index with diabetic kidney disease in patients with type 2 diabetes: a cross-sectional study in Chinese population. Front Endocrinol (Lausanne), 2023. 14: p. 1307692.

50. Yashilha, D., et al., Association Between Monocyte-to-High-Density Lipoprotein (HDL) Cholesterol Ratio and Proteinuria in Patients With Type 2 Diabetes Mellitus: A Prospective Observational Study. Cureus Journal of Medical Science, 2023. 15(9).

51. Yay, F., et al., Can immature granulocytes and neutrophil-lymphocyte ratio be biomarkers to evaluate diabetic nephropathy?: A cross-sectional study. J Diabetes Complications, 2024. 38(9): p. 108807.

52. Zahid Kocak, M., et al., Mean platelet volume to lymphocyte ratio as a novel marker for diabetic nephropathy. Journal of the College of Physicians and Surgeons Pakistan, 2018. 28(11): p. 844-847.

53. Zhang, R., et al., Increased neutrophil count Is associated with the development of chronic kidney disease in patients with diabetes. J Diabetes, 2022. 14(7): p. 442-454.

54. Zeng, G., et al., Relationship of the Neutrophil-Lymphocyte Ratio with All-Cause and Cardiovascular Mortality in Patients with Diabetic Kidney Disease: A Prospective Cohort Study of NHANES Study. J Multidiscip Healthc, 2024. 17: p. 2461-2473.

55. Zhang, J., et al., Effects of neutrophil-lymphocyte ratio on renal function and histologic lesions in patients with diabetic nephropathy. Nephrology (Carlton), 2019. 24(11): p. 1115-1121.

56. Zhang, D., S. Ye, and T. Pan, The role of serum and urinary biomarkers in the diagnosis of early diabetic nephropathy in patients with type 2 diabetes. PeerJ, 2019. 7: p. e7079.

**Table S3.** Quality evaluation of the eligible studies with Newcastle–Ottawa scale(Cohort study).

| Study | Selection | | | | Comparability | | Outcome | | |
| --- | --- | --- | --- | --- | --- | --- | --- | --- | --- |
|  | Representative-ness | Selection of  non-exposed | Ascertainment  of exposure | Outcome not present at start | Comparability on most important factors | Comparability on other risk factors | Assessment of outcome | Long enough follow-up (median≥1 year) | Adequacy  (completeness) of follow-up |
| Akase 2020[1] | * | * | * | * | - | - | * | * | * |
| Cheng 2020[2] | * | * | * | * | - | - | * | * | * |
| Cardoso 2021[3] | * | * | * | * | - | * | * | * | * |
| Sato 2017[4] | * | * | * | * | - | * | * | * | * |
| Wheelock 2018[5] | * | * | * | * | - | - | * | * | * |
| Xie 2024[6] | * | * | * | * | - | - | * | * | * |
| Yashilha 2023[7] | * | * | * | * | - | - | * | - | * |
| Zhang 2022[8] | * | * | * | * | - | - | * | * | * |
| Zeng 2024[9] | * | * | * | * | - | - | * | * | * |

*indicates criterion met; - indicates significant of criterion not met.

**Table S4.** Quality evaluation of the eligible studies with Newcastle–Ottawa scale(case-control).

| Study | Selection | | | | Comparability | | Outcome | | |
| --- | --- | --- | --- | --- | --- | --- | --- | --- | --- |
|  | Proper selection | Representative-ness | Selection of  non-exposed | Determination of control group | Comparability on most important factors | Comparability on other risk factors | Assessment of outcome | Assessment of outcome in non-exposed | Nonresponse rate |
| Akbas 2014[1] | * | * | * | * | - | * | * | * | * |
| Assulyn 2020[2] | * | - | * | * | - | - | * | * | * |
| Bhattacharyya 2021[3] | * | * | * | * | - | * | * | * | * |
| Bloch 2020[4] | * | * | * | * | - | - | * | * | * |
| Chen 2024[5] | * | * | * | * | - | - | * | * | * |
| Chollangi 2023[6] | * | * | * | * | - | - | * | * | * |
| Chong 2022[7] | * | - | * | * | - | - | * | * | * |
| Ciray 2015[8] | * | * | * | * | - | - | * | * | * |
| Demirtas 2015[9] | * | * | * | * | - | - | * | * | * |
| Fang 2024[10] | * | * | * | * | - | - | * | * | * |
| Gao 2024[11] | * | - | * | * | - | * | * | * | * |
| Guo 2022[12] | * | * | * | * | - | - | * | * | * |
| Gupta 2018[13] | * | * | * | * | - | - | * | * | * |
| Gurmu 2022[14] | * | * | * | * | - | - | * | * | * |
| Huang 2015[15] | * | * | * | * | - | - | * | * | * |
| Huang 2017[16] | * | * | * | * | - | - | * | * | * |
| Huang 2020[17] | * | * | * | * | - | - | * | * | * |
| Ibrahim 2024[18] | * | * | * | * | - | - | * | * | * |
| Jaaban 2021[19] | * | * | * | * | - | - | * | * | * |
| Jayashree 2023[20] | * | * | * | * | - | - | * | * | * |
| Kahraman 2016[21] | * | * | * | * | - | - | * | * | * |
| Kamrul-Hasan 2021[22] | * | * | * | * | - | - | * | * | * |
| Kawamoto 2019[23] | * | * | * | * | - | - | * | * | * |
| Kaya 2019[24] | * | * | * | * | - | - | * | * | * |
| Khandare 2017[25] | * | * | * | * | - | * | * | * | * |
| Kocak 2020[26] | * | * | * | * | - | - | * | * | * |
| Li 2022[27] | * | * | * | * | - | * | * | * | * |
| Li 2023[28] | * | * | * | * | - | - | * | * | * |
| Li 2023[29] | * | - | * | * | - | - | * | * | * |
| Li 2024[30] | * | * | * | * | - | - | * | * | * |
| Li 2024[31] | * | * | * | * | - | - | * | * | * |
| Liu 2024[32] | * | * | * | * | - | - | * | * | * |
| Long 2024[33] | * | * | * | * | - | - | * | * | * |
| Mattared 2019[34] | * | * | * | * | - | - | * | * | * |
| Onalan 2019[35] | * | * | * | * | - | - | * | * | * |
| Rakesh 2024[36] | * | * | * | * | - | - | * | * | * |
| Singh 2022[37] | * | * | * | * | - | - | * | * | * |
| Subramani 2023[38] | * | * | * | * | - | - | * | * | * |
| Suvarna 2023[39] | * | * | * | * | - | - | * | * | * |
| Taslamacioglu 2023[40] | * | * | * | * | - | - | * | * | * |
| Tutan 2023[41] | * | * | * | * | - | - | * | * | * |
| Wang 2021[42] | * | * | * | * | - | - | * | * | * |
| Xi 2021[43] | * | * | * | * | - | * | * | * | * |
| Yan 2024[44] | * | * | * | * | - | - | * | * | * |
| Yay 2024[45] | * | * | * | * | - | - | * | * | * |
| Zeng 2022[46] | * | * | * | * | - | - | * | * | * |
| Zhang 2019[47] | * | * | * | * | - | * | * | * | * |
| Zhang 2019[48] | * | * | * | * | - | - | * | * | * |

*indicates criterion met; - indicates significant of criterion not met.
