# Supplementary figures and images for: Association of immune-inflammation indexes with incidence and prognosis of diabetic nephropathy: a systematic review and meta-analysis
Source: Front Endocrinol (Lausanne). 2025 Aug 18;16:1532682. doi: 10.3389/fendo.2025.1532682 (PMC12399399; doi:10.3389/fendo.2025.1532682)

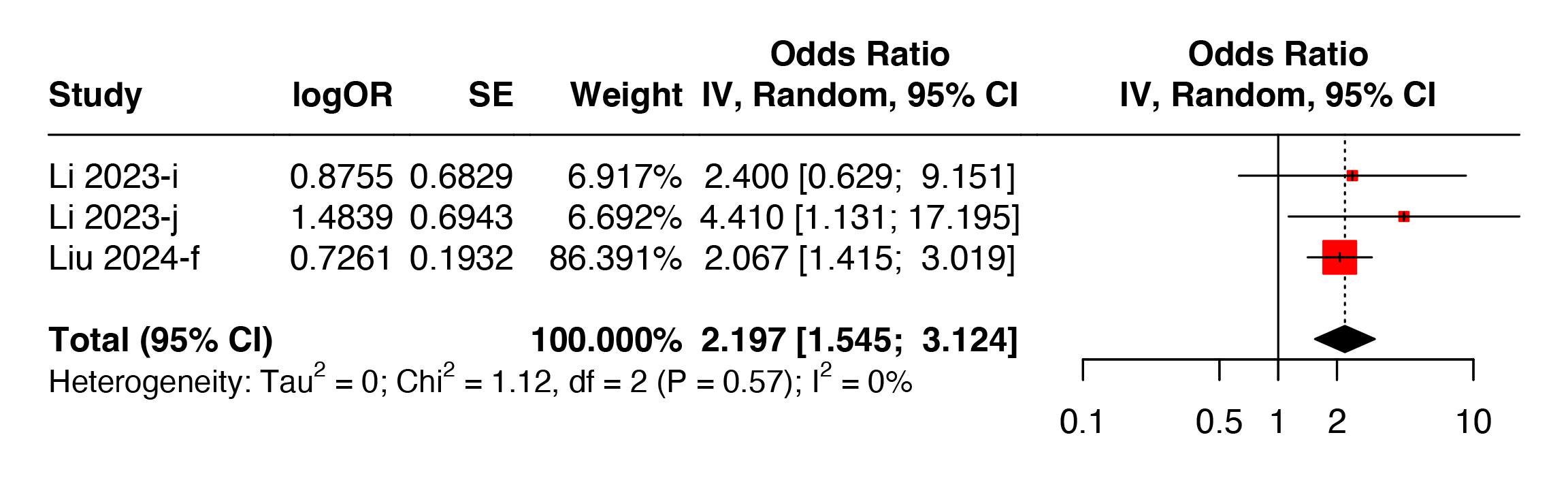

Supplement: Supplementary Figure 1 — Forest plots showing the outcomes for incidence of DN in high SIRI and low SIRI. SIRI, Systemic Inflammation Response Index. [file Image1.tif]

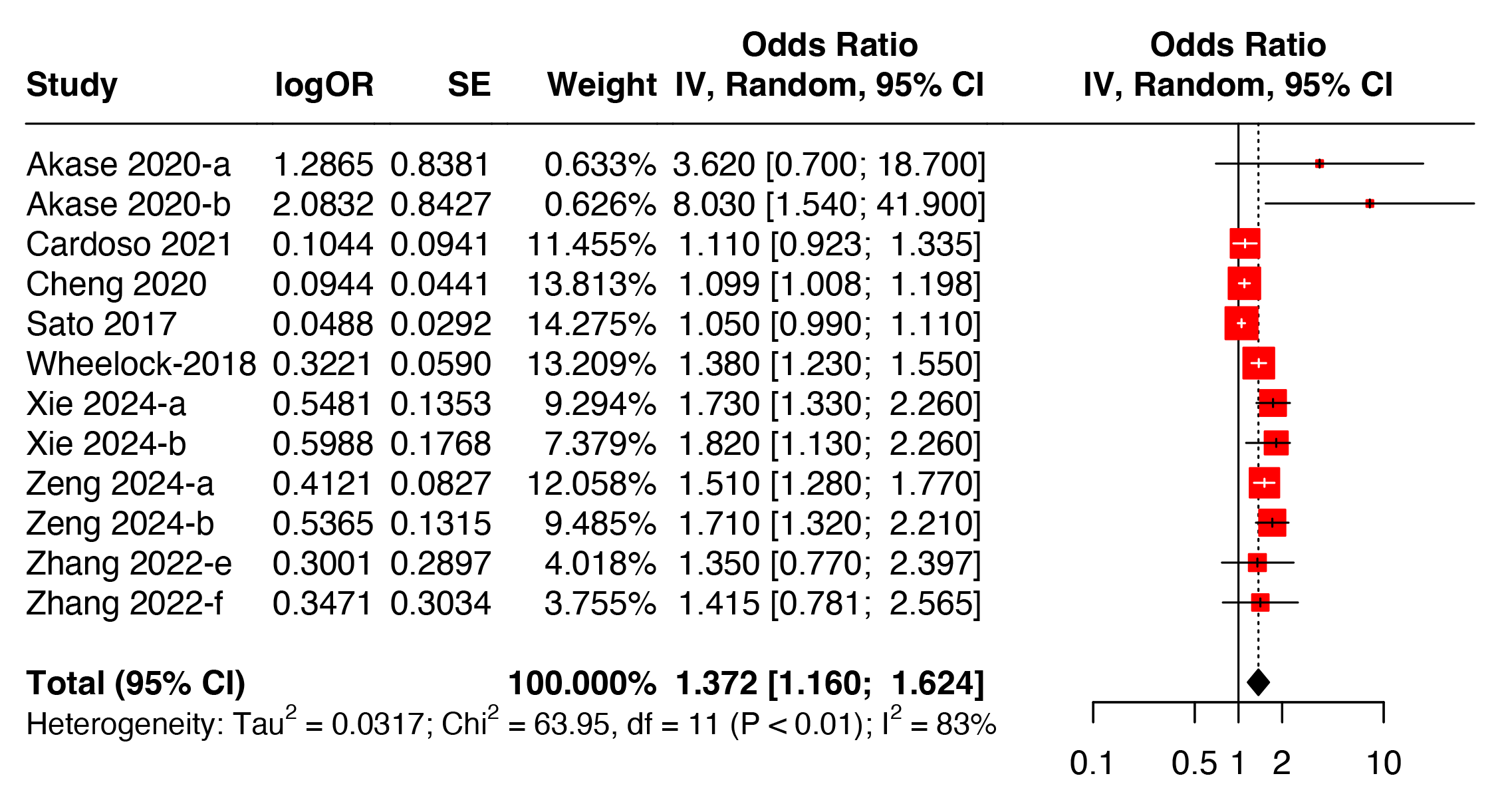

Supplement: Supplementary Figure 2 — Forest plots showing the outcomes for prognosis of DN in high NLR and low NLR. [file Image2.tif]

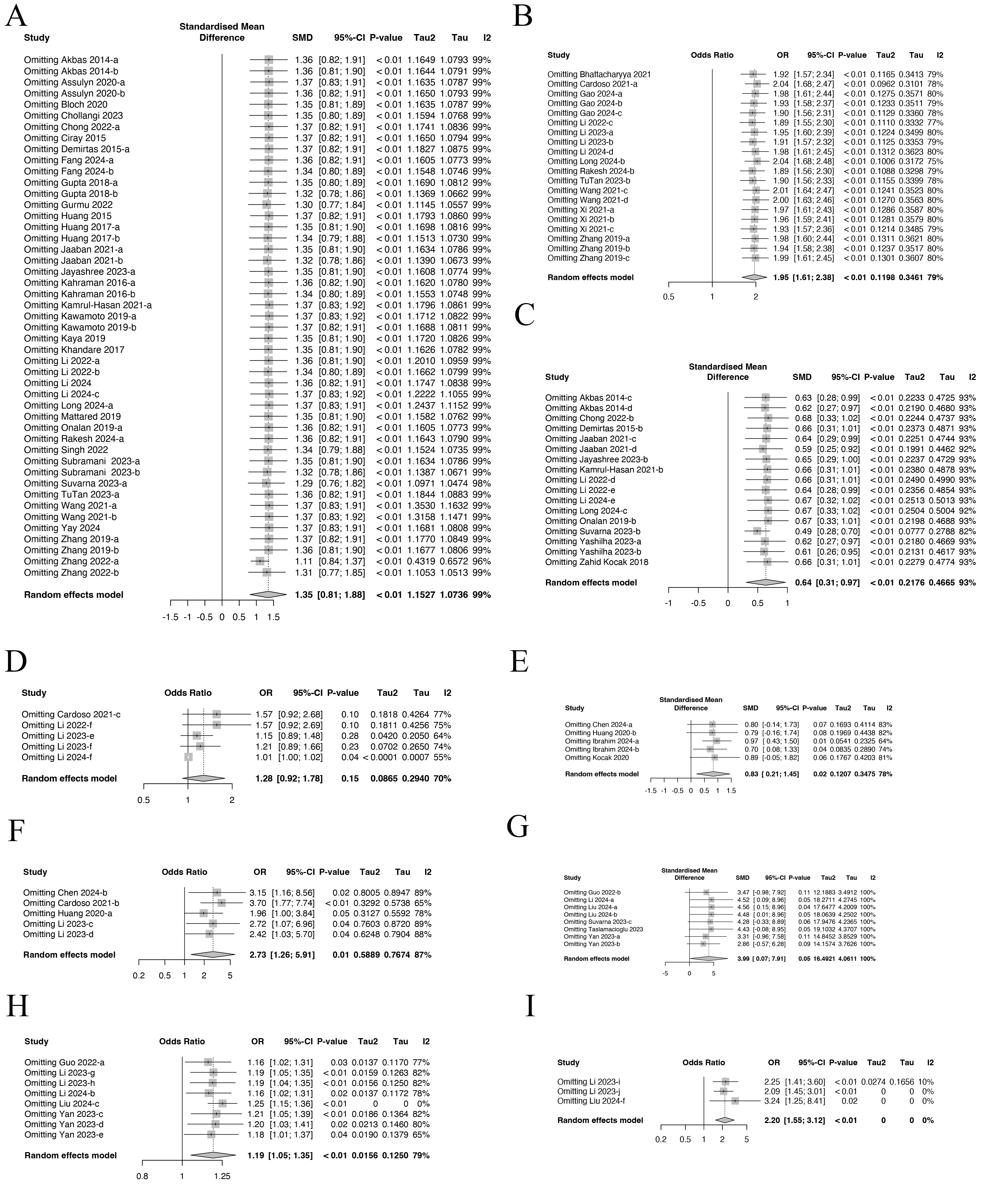

Supplement: Supplementary Figure 3 — The sensitive analysis of selected studies. (A) NLR levels in DN patients;(B) incidence of DN in high NLR and low NLR;(C) PLR levels in DN patients;(D) incidence of DN in high PLR and low PLR;(E) MLR levels in DN patients; (F) incidence of DN in high MLR and low MLR;(G) SII levels in DN patients;(H) incidence of DN in high SII and low SII;(I) incidence of DN in high SIRI and low SIRI [file Image3.tif]

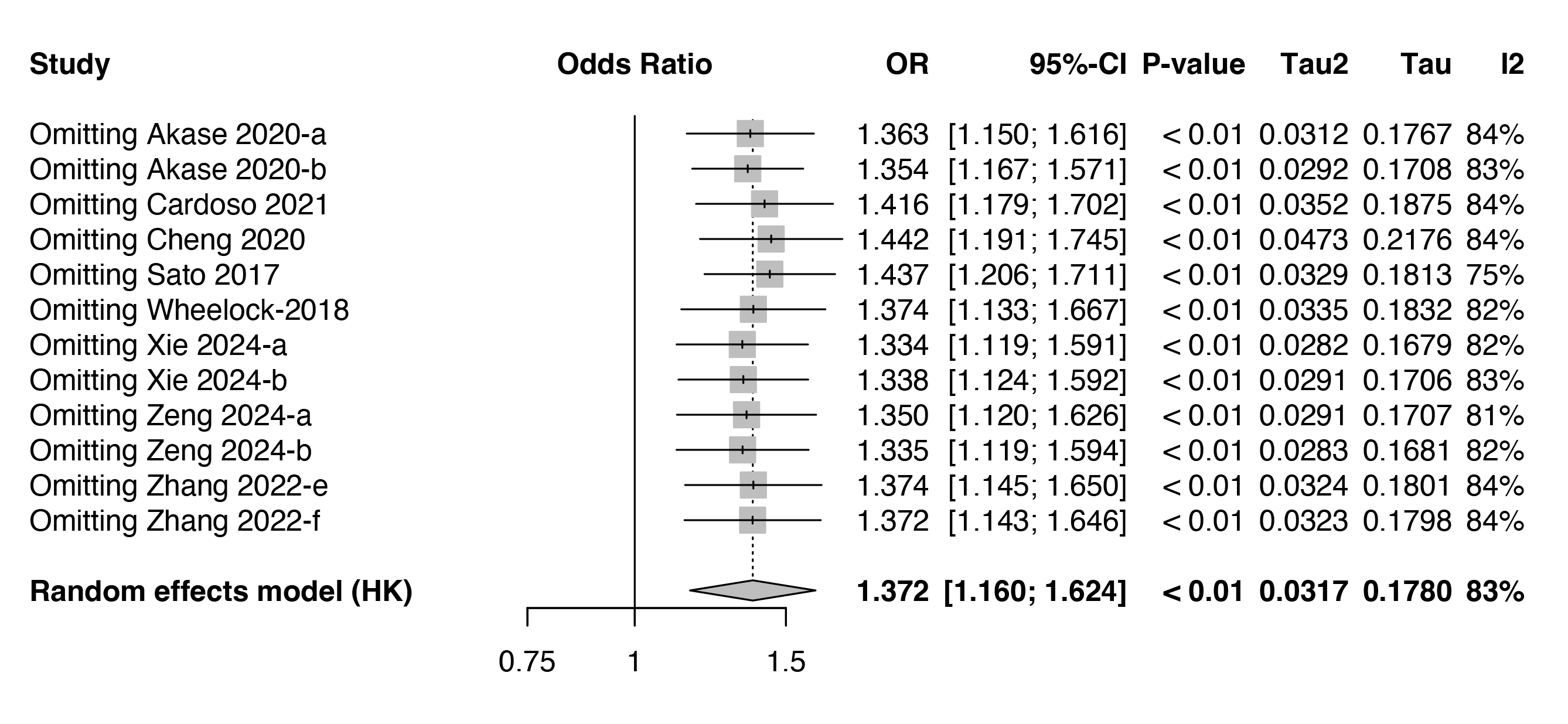

Supplement: Supplementary Figure 4 — The sensitive analysis of the prognosis of DN in high NLR and low NLR. [file Image4.tif]

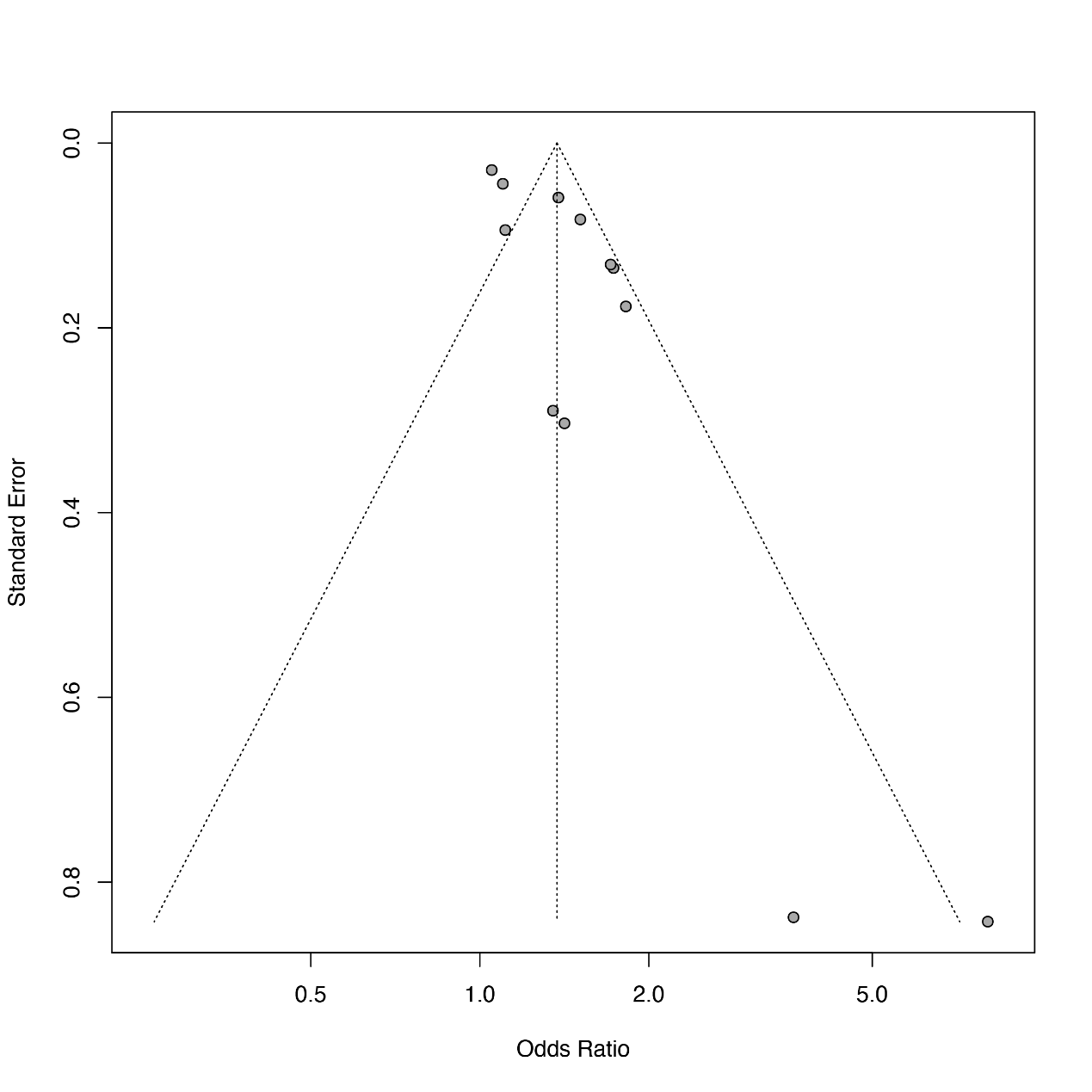

Supplement: Supplementary Figure 5 — Funnel plot of publication bias between NLR and DN prognosis. [file Image5.tif]
